# Phylogenetic study of the genus *Sternolophus* Solier (Coleoptera, Hydrophilidae) based on adult morphology

**DOI:** 10.3897/zookeys.712.14085

**Published:** 2017-10-31

**Authors:** Hiva Nasserzadeh, Helen Alipanah, Ebrahim Gilasian

**Affiliations:** 1 Department of Limnology and Bio-Oceanography, University of Vienna, Althanstrasse 14, A-1090 Vienna, Austria; 2 Insect Taxonomy Research Department, Iranian Research Institute of Plant Protection, Tehran, Iran

**Keywords:** biogeography, cladistic analysis, diversification, species groups, water scavenger beetles

## Abstract

The phylogeny of the hydrophilid genus *Sternolophus* Solier, 1834 was examined in this study using 60 morphological adult characters, eight of them continuous and 52 discrete. The cladistic analysis resulted in a single most parsimonious tree with two major subclades corresponding, respectively, to species previously assigned to the subgenera *Sternolophus* s. str. Solier and *Neosternolophus* Zaitzev, although they are not re-instated. The species groups *S. angolensis* (Erichson, 1843) and *S. solieri* Castelnau, 1840 are recovered as monophyletic. The biogeography and diversification of the species of *Sternolophus* are briefly discussed.

## Introduction

The genus *Sternolophus* Solier, 1834 is widely distributed in the tropics of the Old World, with only few species occurring in the temperate zones. In a recent taxonomic revision of the genus by Nasserzadeh and Komarek (2017), the number of species was increased from nine (Hansen 1999) to 17.

The phylogeny of *Sternolophus* has been poorly studied. Zaitzev (1909) split the genus into two subgenera, *Sternolophus* s. str. Solier, 1834 and *Neosternolophus* Zaitzev, 1909. His classification was based on the absence or presence of an emargination on the anterior clypeal margin. Although this subdivision was accepted by Orchymont (1919), this author considered the length of the spine on the metaventrite a more significant character. Smetana (1980) elevated *Neosternolophus* to generic rank based on the emargination of the anterior clypeal margin, but this change was later opposed by Hansen (1991). This subgeneric division was also rejected by Watts (1989) based on the wide inter- and intraspecific variation of the mentioned character within the Australian species. The phylogenetic relationships of *Sternolophus* species were also studied by Hansen (1991), Short (2010), Short and Fikáček (2013) and Toussaint et al. (2017), although these studies (with the exception of Short 2010) are mainly focused either on family- and tribe-level relationships (Hansen 1991; Short and Fikáček 2013) or had a biogeographic focus (Toussaint et al. 2017). Short (2010) included seven species of *Sternolophus* in his analysis of the subtribe Hydrophilina which resulted in the monophyly of the subgenus Sternolophus s. str. and the lack of resolution for species of *Neosternolophus*.

Nasserzadeh and Komarek (2017) suggested changes to the subgeneric classification, and proposed two new species groups (the groups *S. angolensis* (Erichson, 1843) and *S. solieri* Castelnau, 1840) based on highest morphological similarity and without including a phylogenetic approach. These authors considered *S. angolensis*, *S. inconspicuus* (Nietner, 1856), *S. mundus* (Boheman, 1851) and *S. solitarius* Nasserzadeh and Komarek, 2017 as members of the *angolensis* group, and placed *S. angustatus* (Boheman, 1851), *S. elongatus* Schaufuss, 1883, *S. mandelai* Nasserzadeh and Komarek, 2017, *S. rufipes* (Fabricius, 1792), and *S. solieri* in the *solieri* group. They left the remaining species (*S. australis* Watts, 1989, *S. decens* Zaitzev, 1909, *S. immarginatus* Orchymont, 1911, *S. insulanus* Nasserzadeh and Komarek, 2017, *S. jaechi* Nasserzadeh and Komarek, 2017, *S. marginicollis* (Hope, 1841), and *S. prominolobus* Nasserzadeh and Komarek, 2017) ungrouped.

Here the first comprehensive phylogenetic analysis of the genus *Sternolophus* is provided, based on a cladistics analysis of adult morphological characters. Considering the phylogenetic results, the biogeography and diversification of the species are briefly discussed.

## Materials and methods

**Taxon sampling.** More than 4000 specimens in all the 17 species of *Sternolophus* were studied as ingroup, and *Hydrochara flavipes*, belonging to the tribe Hydrophilini, was included as outgroup. A total of 271 specimens were measured. The specimens were obtained on loan from the following institutions and collections:

**AEZS** coll. A. Short, University of Kansas, Lawrence, KS, USA

**CBSU** Collection of Department of Biology, Shiraz University, Iran

**HMIM** Hayek Mirzayans Insect Museum, Tehran, Iran

**ISNB** Institut Royal des Sciences Naturelles de Belgique, Bruxelles, Belgique

**MNHN** Muséum National d’Histoire Naturelle, Paris, France

**MNHUB** Museum der Alexander Humboldt Universität, Berlin, Germany

**NHML** Natural History Museum, London, UK

**NMB** Naturhistorisches Museum Basel, Basel, Switzerland

**NMW** Naturhistorisches Museum Wien, Vienna, Austria

**NRM** Swedish Museum of Natural History, Stockholm, Sweden

**OUMNH** Oxford University Museum of Natural History, UK

**SAMA** South Australian Museum, Adelaide, Australia

**SMTD** Staatliches Museum für Tierkunde, Dresden, Germany

**ZMUC** Zoological Museum University of Copenhagen, Denmark

The examined specimens are listed in Appendix 1. The specimens were selected according to: 1) geographical distribution, 2) morphological variation, and 3) status as type specimens.

**Figures 1–13.**
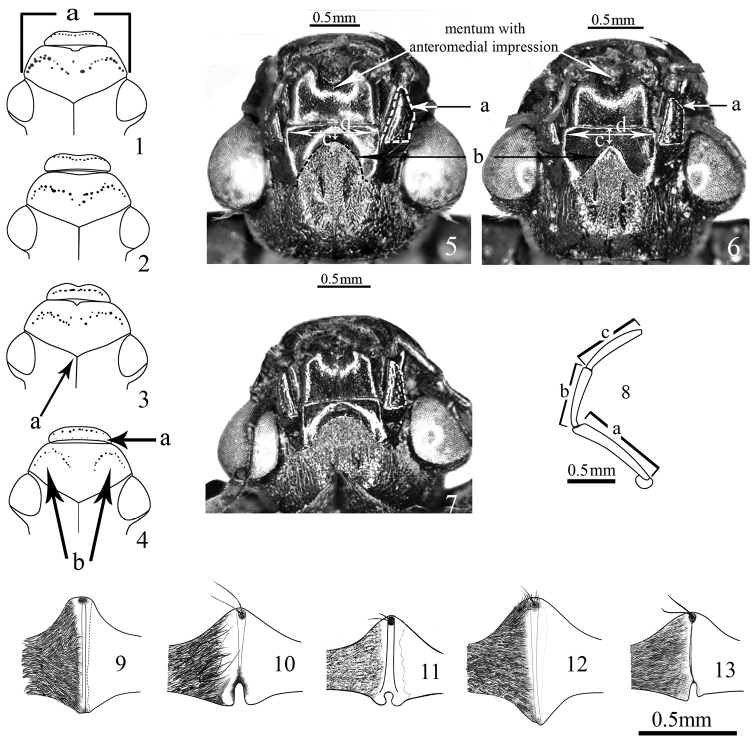
**1–4** Dorsal view of head **1** *Sternolophus acutipenis* **a** width of clypeus at anterior margin of eyes **2** *S. jaechi* **3** *S. marginicollis* **a** centre of frontoclypeal suture **4** *S. solieri* **a** deeper punctures near the basal margin of labrum **b** paired antero-lateral groups of punctures on the clypeus (Nasserzadeh and Komarek 2017) **5–7** Ventral view of head **5** *Sternolophus acutipenis* **6** *S. angustatus* **7** *S. decens* **a** maxilla **b** pubescent area on submentum **c** bare area of submentum **d** base of mentum **8** Maxillary palpus of *Sternolophus acutipenis* **a–c** length of palpus segments (Nasserzadeh and Komarek 2017) **9–13** Prosternal carina **9** *Sternolophus acutipenis* **10** *S. angustatus* **11** *S. decens* **12** *S. jaechi* **13** *S. solieri* (Nasserzadeh and Komarek 2017).

**Figures 14–21.**
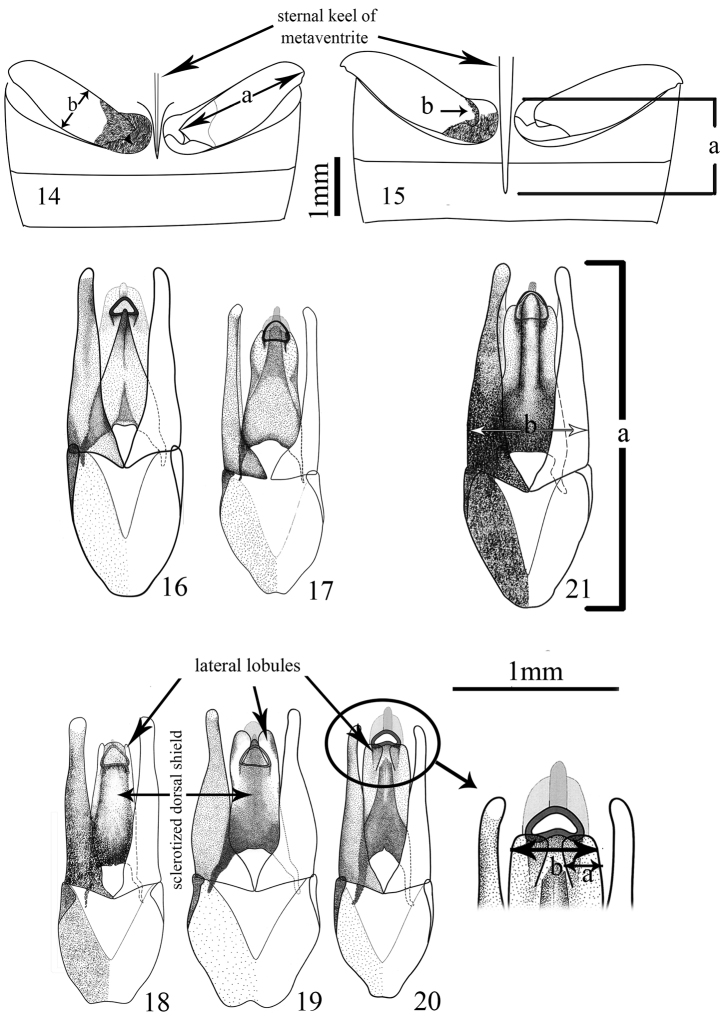
**14–15** Hind femur with the spine on metaventrite **14** *Sternolophus acutipenis* **a** length of femur **b** widest part of hind femur **15** *S. mandelai* **a** length of spine **b** basal pubescent area (modified from Nasserzadeh and Komarek 2017) **16–21** Dorsal view of aedeagus **16** *Sternolophus acutipenis* **17** *S. angolensis* **18** *S. angustatus* **19** *S. immarginatus* **20** *S. marginicollis* **a** lateral lobules at widest part of median lobe **b** total width of median lobe on apical portion of the sclerotized dorsal shield **21** *S. solitarius* **a** length **b** widest part of the parameres (modified from Nasserzadeh and Komarek 2017).

**Preparation for morphological studies.** To study the male genitalia, the aedeagus was extracted and macerated in lactic acid for at least four days to become hydrated and cleared before examination. Bursa copulatrix, spermatheca, and spermathecal gland were also dissected (for details see Nasserzadeh et al. 2005) and mounted in DMHF or Euparal on transparent cards and pinned below the associated specimens. Morphological data for each species were obtained using a stereomicroscope (Zeiss Stemi SV11). Measurements were made through a micrometric eyepiece and presented in figures 1, 8, 14−15, 20−21. Line drawings of characters were adapted from Nasserzadeh and Komarek (2017). Photographs were taken using a 650D Canon digital camera.

**Character selection and coding.** Character selection and character state definition follow Smetana (1980), Nasserzadeh et al. (2005) and Nasserzadeh and Komarek (2017). A total of 60 characters (eight continuous and 52 discrete) was selected and scored from zero to 59 (see Table 1). Eight continuous characters involving ranges and ratios were treated as such, avoiding the use of *ad hoc* methods to establish ranges (Goloboff et al. 2008). Discrete characters contained 45 binary and seven multistate. Characters 0, 2−6, and 8−45 correspond to the external morphology, characters 1, 7 and 46−55 were derived from the aedeagus, and characters 56−59 were coded from the female genital membranous tube. Characters and character state compositions approach the logic of neomorphic and transformational pattern as indicated by Sereno (2007). There are no missing characters in the data matrix, and the inapplicable characters were coded as ‘?’ (Appendix 2).

**Table 1.** List of morphological characters, character states, and codes.

Codes	List of characters and character states
**Continuous characters**	
0	Average length of body in millimeters.
1	Average length of aedeagus in millimeters (Fig. 15a).
2	Ratio width of head (from outer lateral margin of eyes) / width of clypeus in anterior margin (connecting with labrum) in males.
3	Ratio width of head in outer margin of eyes / length of clypeus (from the centre of frontoclypeal suture (Fig. 3a) to anterior margin of clypeus).
4	Ratio average length of body / average length of aedeagus.
5	Length of hind femur (Fig. 13a) / widest part (Fig. 13b).
6	Ratio distance of bare area between the apical angle of the pubescent part of submentum to the base of mentum (Figs 5c, 6c) / width of anterior margin of submentum (connecting to the mentum) (Figs 5d, 6d).
7	Ratio length of aedeagus (Fig. 15a)/width (widest part of the parameres) (Fig. 15b).
**Discrete characters**	
*External body morphology*	
8	Lateral sides of body: (0) rather parallel; (1) rather rounded.
9	Body in lateral view: (0) distinctly convex; (1) moderately convex.
10	Femora with basal hydrofuge pubescent: (0) absent; (1) present.
11	If femora pubescent basally, pubescence distribution on hind femur: (0) very narrow, in anterior part of femur connecting with coxa, sometimes slightly extended marginally to the connecting border with trochanter (Fig. 14b); (1) more expanded, covering a wider area from attachment part of femur to coxa posteriorly toward trochanter (Fig. 13).
12	Coloration of legs in comparison with ventrites: (0) unicolored; (1) not unicolored.
13	Coloration of femur: (0) uniformly black to rufous; (1) not uniformly colored, femur distinctly darker proximally and lighter distally, rufo-testaceous to rufous.
14	Irregular transversal row of 11–13 deep punctures on medial part of the labrum: (0) absent; (1) present.
15	Few deeper punctures near the basal margin of labrum (Fig. 4a): (0) absent; (1) present.
16	Length of the rufous to testaceous coloration on the anterior part of labrum /length of labrum: (0) ¼ to ⅙; (1) ½ to ⅓.
17	Paired and irregularly distributed antero-lateral groups of punctures on the clypeus (Fig. 4b): (0) semicircular (Figs 1–3); (1) arc-shaped (Fig. 4).
18	The paired antero-lateral groups of punctures on the clypeus separated: (0) narrowly (narrower than 1/6 width of clypeus at anterior margin of eyes); (1) widely (wider than 1/5 width of clypeus at anterior margin of eyes).
19	Anterior margin of clypeus: (0) entire (Fig. 4); (1) sinuated/emarginated medially (Figs 1–3).
20	If anterior margin of clypeus emarginated or sinuated medially: (0) sinuated smoothly (Fig. 2); (1) weakly emarginated; (2) distinctly emarginated (Fig. 3); (3) strongly and widely emarginated (Fig. 1).
21	Apex of fourth maxillary palpomere: (0) without infuscation; (1) distinctly darkend.
22	Length of maxillary palpus (Fig. 7) /width of clypeus in anterior margin of eye: (0) short (0.8); (1) almost equal (1.0); (2) moderately long (1.2−1.3); (3) long (1.4).
23	Mentum with anteromedial impression: (0) absent; (1) present (Figs 5–7).
24	If mentum with anteromedial impression, the pubescent area of submentum: (0) triangular-shape, lateral sides more straight (Fig. 6); (1) semicircular-shape, lateral sides more rounded (Fig. 7); (2) belly-shape/domical-shape, rounded lateral sides (Fig. 5)
25	Outer lateral margin of maxilla: (0) rounded, without projection; (1) not rounded, more or less straight, with or without a projection (Figs 5–7).
26	If lateral margin of maxilla is straight: (0) no projection on lateral margin is recognizable (Fig. 7); (1) a distinct projection is recognizable (Figs 5–6).
27	If lateral margin of maxilla bears a distinct projection: (0) it is located approximately on anterior third (Fig. 6); (1) it is located approximately on medial portion (Fig. 5).
28	Scattered deep punctures on pronotum: (0) absent; (1) present.
29	Mesal edge of prosternal carina: (0) sharp (Figs 9, 10, 12, 13); (1) blunt (Fig. 11).
30	Deep or weak division on posterior end of mesal edge of prosternal carina: (0) absent (Figs 8, 11); (1) present (Figs 10, 11, 13).
31	If mesal edge of carina not divided and knob-like, posterior protrusion between procoxae: (0) absent (Fig. 7); (1) present (Fig. 10).
32	If mesal edge of carina divided on posterior end, the division is: (0) deep with a notch (Fig. 9); (1) more or less weak and without a deep notch (Fig. 12).
33	Number of longitudinal series of punctures on the elytra: (0) four; (1) five.
34	If the number of longitudinal series of punctures on the elytra is four, irregular punctures between last lateral series 4 and elytral margin: (0) absent; (1) present.
35	If number of longitudinal series of punctures on the elytra is four and irregular punctures between last lateral series and elytral margin present, the width of punctures in interspace of lateral margin of elytra (between lateral series and elytral margin): (0) about ¾ or more; (1) about ½; (2) about ⅓ or less.
36	If irregular punctures between lateral series 4 and elytral margin reaching 1/2 width of interspace, irregular punctures distributed: (0) densely; (1) loosely.
37	Length of spine on metaventrite: (0) short, never reaching anterior margin of first ventrite (Fig. 14); (1) long, exceeding anterior margin of first ventrite (Fig. 15).
38	If length of spine on metaventrite long, spine: (0) straightly elongated almost in parallel to the ventral side; (1) slightly and gradually bend upward distally toward posterior end.
39	If the spine of metaventrite short, spine at posterior end (or apex): (0) not sharp/pointed, not bent ventrally; (1) sharp and slightly bent ventrally.
40	If the spine of metaventrite short, spine: (0) reaching mid-length of 1^st^ ventite or shorter (Fig. 12); (1) exceeding mid-length of 1^st^ ventrite (Fig. 13).
41	If the spine of metaventrite long, spine: (0) not reaching mid-length of 2^nd^ ventrite (1) hardly reaching mid-length of 2^nd^ ventrite; (2) exceeding mid-length of 2^nd^ ventrite and extending to 3/4 length of ventrite 2; (3) reaching anterior margin of 3^rd^ ventrite.
42	Sternal keel of metaventrite: (0) slim, almost as wide as the spine of metaventrite at mid-length (Fig. 14); (1) wide, distinctly wider than the spine on metaventrite at mid-length (Fig. 15).
43	Abdominal ventrite 5 hydrofuge pubescence: (0) uniform; (1) with a glabrous posteromedian area.
44	Apical margin of ventrite 5: (0) entire; (1) emarginated.
45	Male claw of fore leg: (0) weakly curved and short; (1) strongly curved and distally elongated.
*Aedeagus morphology*	
46	Inner and outer lateral margins of paramere on anterior half: (0) without distinct curvature and straight (Fig. 17); (1) with curvature, i.e. width of paramere changes from mid-length toward the apex. (Figs 18–21).
47	If paramere with curvature in lateral margins on anterior half: (0) outer lateral margin concave at about mid-length (Fig. 20); (1) outer lateral margin concave at about apical third (Figs 16, 18, 19, 21).
48	If paramere with outer lateral margin concave at about apical third: (0) the posterior ⅔ smoothly and widely convex with no impression (Fig. 19); (1) a weak curvature projected lateromedially (just before the apical third) (Figs 16, 18, 21).
49	If outer lateral margin of paramere concave at about apical third without a smooth convex curve, the apex of paramere: (0) clavate (Figs 18, 21); (1) not clavate (Fig. 16).
50	Sclerotized dorsal shield of median lobe of aedeagus: (0) without sharp anterior carina; (1) with sharp anterior carina (Fig. 16).
51	Sclerotized dorsal shield of median lobe of aedeagus: (0) flat to subcylindrical (Figs 17, 18, 21); (1) tectiform (Figs 16, 19, 20).
52	Lateral lobules of median lobe of aedeagus: (0) absent (Fig. 16); (1) present.
53	If lateral lobules of median lobe of aedeagus present, lateral lobule at widest part (Fig. 20a) / total width of the median lobe on apical portion of the sclerotized dorsal shield (Fig. 20b): (0) less than 2/10 (lobules with small size) (Fig. 18); (1) almost 3/10 (lobules with moderate size) (Figs 17, 21); (2) almost 4/10 (lobules with large size) (Figs 19, 20).
54	If lateral lobules of median lobe of aedeagus present, the sclerotized dorsal shield: (0) without snout-shaped process apically that protrudes between the lateral lobules (Figs 18, 20); (1) with a weak snout-shaped process apically that protrudes between the lateral lobules (Figs 17, 19, 21).
55	If lateral lobules of median lobe of aedeagus present these lobules: (0) not inflated; (1) inflated (Fig. 19).
*Female genital tube morphology*	
56	Connection between bursa copulatrix and ejaculatory duct: (0) lateral; (1) anterior.
57	Connection of spermathecal duct and spermathecal gland to spermathecal bulb: (0) separate; (1) via one joined duct.
58	Length of spermathecal duct/bursa (from apex to common oviduct): (0) less than 1/2; (1) 1/2 to equal; (2) two times longer.
59	Longitudinal rows of small tooth-like spines on the membranous wall of the bursa: (0) absent; (1) present.

**Phylogenetic analysis.** Cladistic analyses were performed on all characters in ‘Tree Analysis using New Technologies’ (TNT) (Goloboff et al. 2008) with ‘traditional’ search based on 5000 replicates, through ‘tree bisection reconnection’ (TBR) branch swapping holding 100 trees by collapsing rule ‘min. length=0’. Discrete characters were treated as unordered, and multistate characters were treated as polymorphic (e.g. [0 1]). The same analysis was performed only on the discrete characters and the consensus tree was obtained using strict and majority-rule methods. An analysis including all continuous and discrete characters was also conducted by retaining suboptimal trees 0.5 steps longer than the most parsimonious tree; the resulting trees were summarized by strict and majority-rule consensus methods.

The synapomorphic characters and character states are mapped on the single most parsimonious cladogram (analysis A). Branch support was calculated by bootstrap (Felsenstein 1985), jack-knife (Farris et al. 1996), and symmetric resampling (Goloboff et al. 2003), with 2000 replicates. Different numbers of replicates (up to 5000) did not affect the results. In resampling analysis, the results of the absolute frequency summarize method was used, which were slightly higher than the analysis using frequency difference.

The consistency and retention indices (Kluge and Farris 1969; Farris 1989) of discrete characters were calculated using PAUP version 4.0b10 (Swofford 2002) (analysis D). All 52 discrete characters were equally weighted, and multistate characters were treated as unordered. Heuristic searches were selected with 20000 random additions followed by branch swapping using TBR and holding a single tree (NCHUCK = 1, CHUCKSCORE = 1) (Alipanah et al. 2010).

## Results

**Figure 22.**
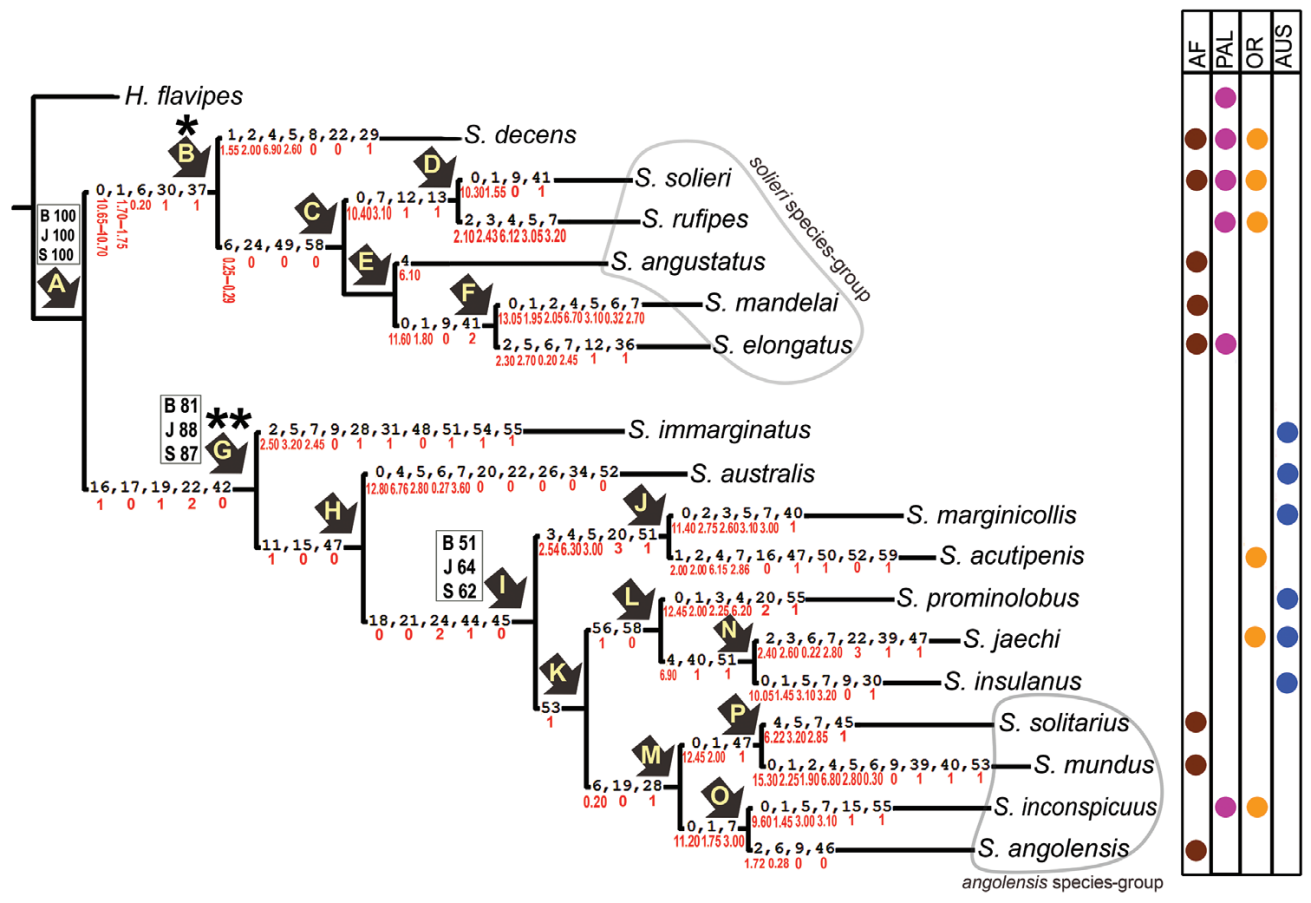
Single most parsimonious tree (146.130 steps) based on 60 morphological characters (52 discrete and 8 continuous). Bootstrap (B), Jackknife (J) and Symmetric (S) support values over 50% are mentioned above the corresponding branches, respectively. The arrows with capital letters indicate the clades. Synapomorphies are shown on the branches, and character states in red. Table on the right shows distribution of the species by region (AF = Afrotropical, PAL = Palaearctic, OR = Oriental, AUS = Australian). The two major clades are marked as (*) and (**) indicating *Sternolophus* s. str. and *Neosternolophus* respectively. Species groups *angolensis* and *solieri* (see Nasserzadeh and Komarek 2017) are shown in closed irregular ovals.

**Figure 23.**
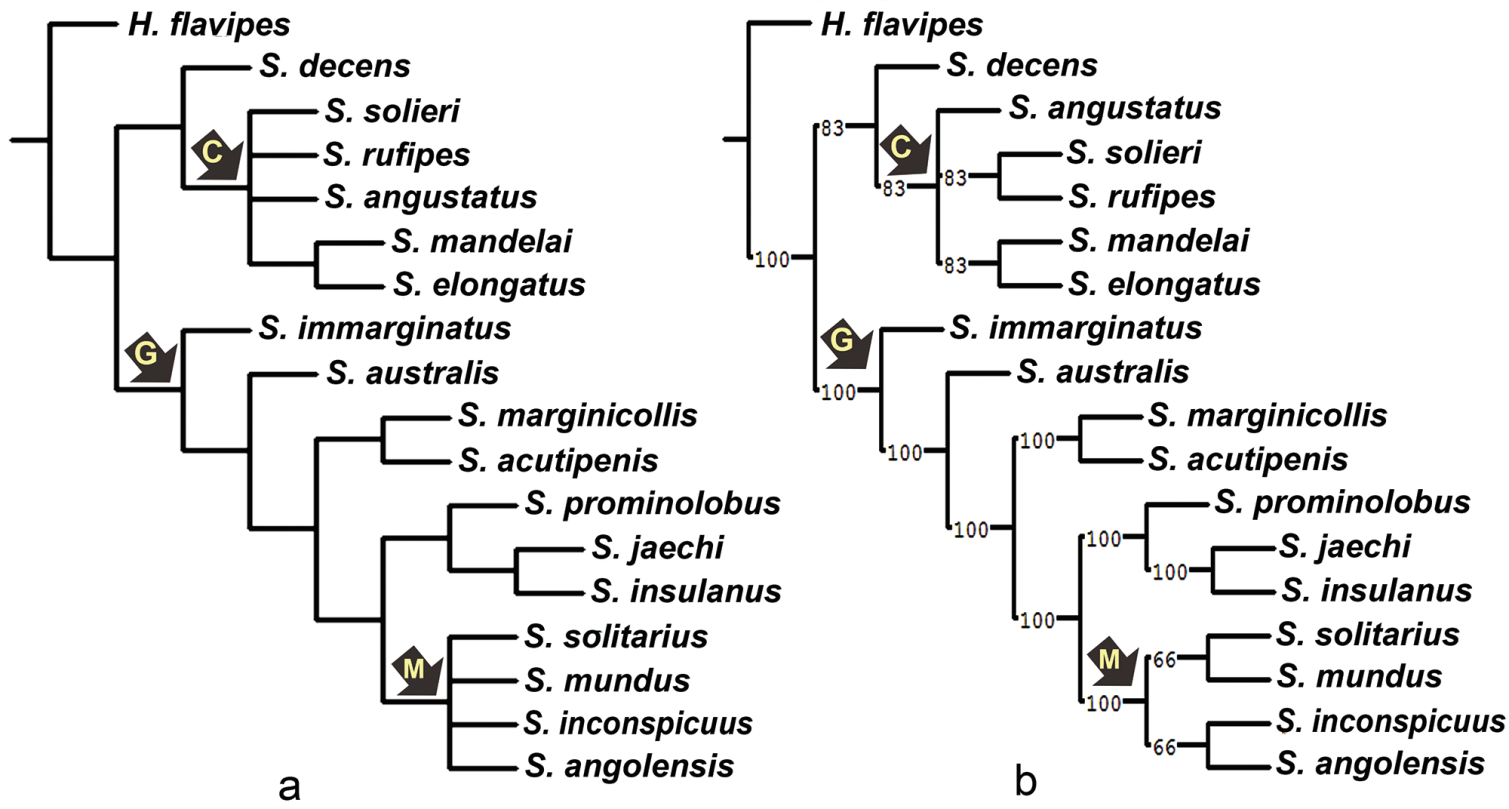
Results of the phylogenetic analysis based on 60 (continuous and discrete) morphological characters, with a suboptimum value of 0.5 step longer **a** strict consensus tree **b** majority-rule consensus tree of six most parsimonious trees (length 146.130), numbers on the branches indicate majority rule support for node. The arrows with capital letters indicate selected clades.

**Figure 24.**
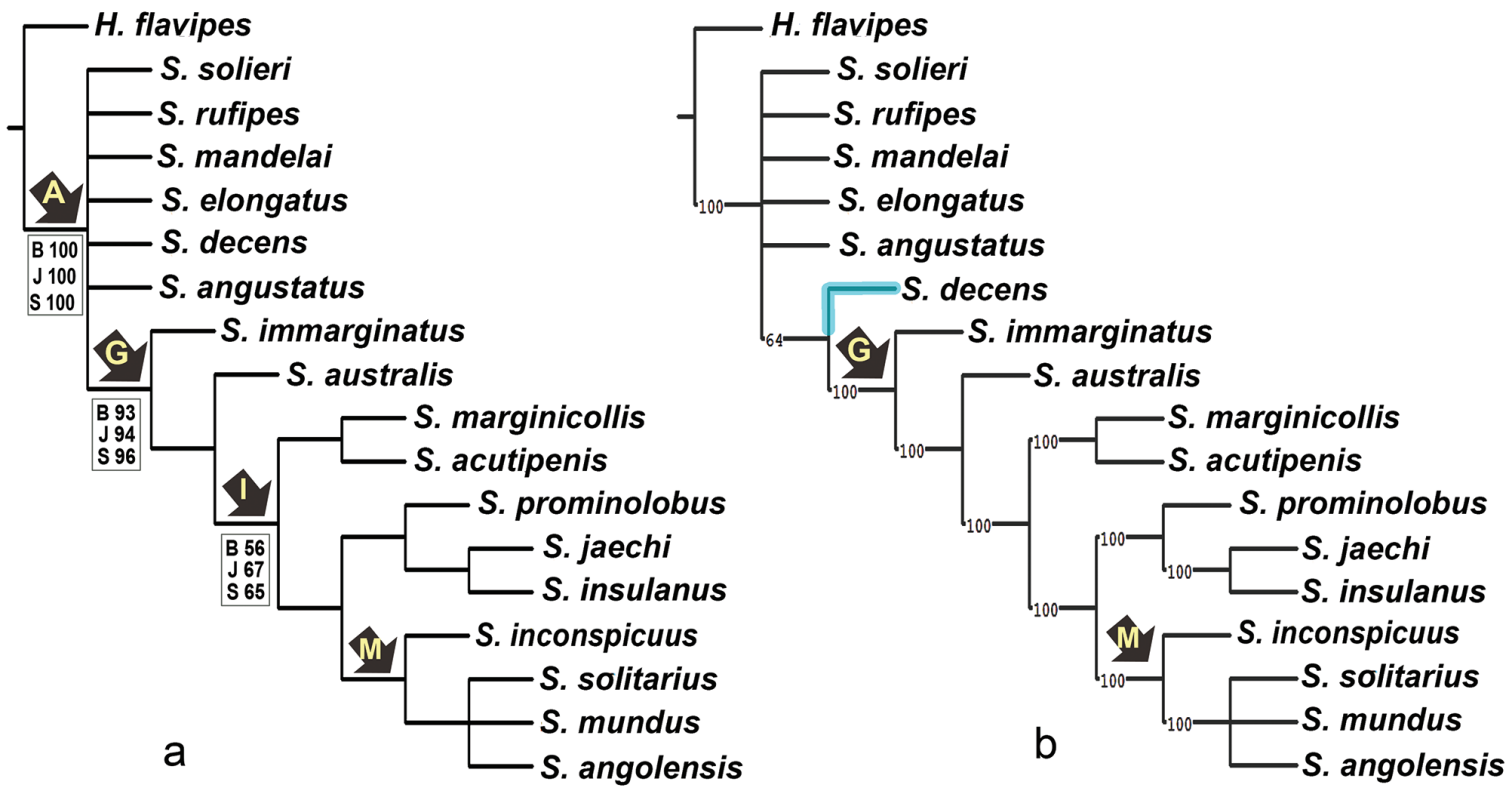
Results of the phylogenetic analysis based on 52 discrete morphological characters. **a** strict consensus tree. Bootstrap (B), Jackknife (J) and Symmetric (S) support values over 50% are mentioned above the corresponding branches **b** majority-rule consensus tree of 36 most parsimonious trees (length 110). Numbers on the branches indicate majority rule support for nodes. Arrows with capital letters indicate selected clades.

The parsimony analysis of all characters (analysis A) resulted in a single most parsimonious tree of 146.130 steps (Fig. 22). When suboptimal trees 0.5 steps longer than the most parsimonious tree were retained (analysis C), six most parsimonious trees were obtained. The consensus of these trees, either using strict or majority-rule methods, was congruent with the single most parsimonious tree from analysis A, except for slight differences in the position of the species within clades C and M (Fig. 23a, b). The analysis of discrete characters only (analysis B) resulted in 36 most parsimonious trees of 110 steps. The consensus trees using both strict and majority-rule methods were different from previous trees in the position of the species in clade B (Fig. 24a, b). Analysis using PAUP on the 52 discrete characters (analysis D) estimated 38 parsimony informative characters, with consistency index (CI) = 0.56 and retention index (RI) = 0.72.

As shown in the single most parsimonious tree obtained with analysis A (Fig. 22), the examined *Sternolophus* species are divided into two major monophyletic clades, B and G, with 6 and 11 species respectively. Clade B contains *S. decens* as sister to clade C that is composed of five species, *S. solieri*, *S. rufipes*, *S. angustatus*, *S. mandelai*, and *S. elongatus*. Clade B is supported by five characters (0: 10.65–10.70, 1: 1.70–1.75, 6: 0.20, 30: 1, 37: 1), although it is weakly supported statistically. Except for the elongated spine on the metaventrite (37: 1), the characters sustaining this clade were homoplastic. The topology of clade B was slightly different in analysis C (Fig. 23), and the clade was not maintained in analysis B, with the six species unresolved in the strict consensus (Fig. 24a), whereas in the majority-rule consensus tree (Fig. 24b) *S. decens* was resolved as sister to clade G in 64% of the cases (24 out of 36 trees).

The monophyly of clade G was well supported in all analyses (Figs 22–24). Monophyly of this clade is supported by the following five synapomorphies: the rufous to testaceous coloration of the labrum exceeding one third of its length (16: 1); the semicircular arrangement of the paired antero-lateral group of punctures on clypeus (17: 0); the presence of an emargination on the anterior margin of clypeus (19: 1); the moderately long maxillary palpus (22: 2); and the slim sternal keel of metaventrite (42: 0). All analyses also agreed in the monophyly of clade I, although with weaker support (Figs 22–24). Five synapomorphies sustain this clade: the narrow distance between paired antero-lateral groups of punctures on the clypeus (narrower than one-sixth of the width of clypeus at anterior margin of eyes) (18: 0); the absence of infuscation on the apex of fourth maxillary palpomere (21: 0); the belly shape of the pubescent area of submentum (24: 2); the presence of an emargination on the apical margin of ventrite 5 (44: 1); and the weakly curved and short male claw on fore leg (45: 0). Based on the results of analysis A (Fig. 22), *S. australis* is sister to clade I, whereas *S. immarginatus* is sister to the clade formed by *S. australis* and clade I. In all analyses, clades K, L, M, and N were found to be monophyletic with the same configuration. These clades are supported by one, two, three, and three synapomorphies, respectively (Fig. 22); however, the position of the four species within clade M was unstable in all analyses.

The comparison of the trees obtained using all characters (Figs 22, 23) with those obtained using only discrete characters (Fig. 24) reveals the influence of continuous characters in the formation of clade B. The exclusion of continuous characters from the analysis causes the species within this clade to collapse in a polytomy (Fig. 24). Clade B is supported by three continuous and two discrete synapomorphies. Similarly, continuous synapomorphies outnumber discrete synapomorphies within clade B, except for clade C with one continuous and three discrete synapomorphies (Fig. 22). The importance of continuous characters in shaping clade B can be explained by the fact that this character set (0 to 7) provides diagnostic features for separating the morphologically very similar species of the *solieri* species group (clade C) (Nasserzadeh & Komarek 2017). In all analyses, the topology of clade G remained consistent except for slight changes in clade M and variable support for clades G and I (Figs 22–24). On the other hand, *Sternolophus decens* was recovered in clade B in five of the six most parsimonious trees obtained using both continuous and discrete characters combined (Fig. 23b), whereas it was sister to clade G in more than 60% of the 36 most parsimonious trees obtained using discrete characters only (e.g., Fig. 24b), showing that the position of this taxon is also highly influenced of continuous characters.

## Discussion

**Taxonomy.** The species formerly included in the subgenera *Sternolophus* s. str. and *Neosternolophus* were recovered into two major subclades, B and G, respectively. However, due to the following considerations, subgeneric status was not re-instated: i) Unreliable topology of clade B in different analyses and absence of support for its monophyly as well as monophyly of the subclades. ii) Questionable position of *S. decens* within clade B. *Sternolophus decens* was included in the subgenus Sternolophus s. str. by Zaitzev (1909), and was found to be closely related to *S. rufipes* and *S. solieri* by Short (2010). However, it was recovered in a monophyletic clade together with *S. marginicollis* (and some unidentified *Sternolophus* species) by Toussaint et al. (2017), which was included in the subgenus Neosternolophus by Zaitzev (1909). In the trees obtained in analyses A and C (Figs 22–23), *S. decens* was recovered as sister to clade C. The species of this clade (*S. solieri*, *S. rufipes*, *S. angustatus*, *S. mandelai* and *S. elongatus*) (Fig. 22) were grouped in the *solieri* species group by Nasserzadeh and Komarek (2017) based on highest morphological similarity. iii) A nearly similar topology was obtained for clade G in the different analyses, all of them including *S. marginicollis*, with strong support. Based on the topology obtained here and those of Short (2010) and Toussaint et al. (2017), we believe that reinstating subgenera within *Sternolophus* is premature and would not reflect the evolutionary history of the genus. Further investigations including larval and molecular characters of as many species of the genus as possible, as well as other techniques such as scanning electron microscopy, are required to resolve its phylogenetic relationships.

Short (2010), in his phylogenetic analysis of the subtribe Hydrophilina based on adult-morphological characters, found evidence for monophyly of the subgenus Sternolophus s. str., but the species formerly grouped in the subgenus Neosternolophus were unresolved and formed a basal polytomy within the genus. In our analysis, on the contrary, strong evidence was found for monophyly of *Neosternolophus*, whereas monophyly of *Sternolophus* s. str. is more questionable for the reasons mentioned above.

Finally, the four species (*S. solitarius*, *S. mundus*, *S. inconspicuus* and *S. angolensis*) grouped by Nasserzadeh and Komarek (2017) as the *angolensis* species group based on morphological similarities, are resolved here as clade M confirming their close relationship, although weakly supported (Fig. 22).

**Biogeography and diversification.** In Figure 22 (right table), clade C consists of the *solieri* species group distributed in the Afrotropical, Palaearctic and Oriental regions. Distribution of *S. decens* overlaps with those of clade D. On the other hand, most members of clade G have an Oriental-Australasian distribution. The exceptions are representatives of the *angolensis* species group, with *S. solitarius*, *S. mundus*, and *S. angolensis* restricted to the Afrotropical Region whereas *S. inconspicuus* is widely distributed in the Oriental Region to the eastern boarder of the Palaearctic Region. *Sternolophus insulanus* and *S. jaechi* are two sister species with insular distribution in the Malay Archipelago (see Appendix 1).

Toussaint et al. (2017) postulated an Afrotropical origin for *Sternolophus*, dispersing toward Australia in the Oligocene/Miocene. There are many New Cenozoic fossil findings of taxa closely related to *Sternolophus* in Europe and North America (e.g. Fikáček et al. 2008, 2010a, 2010b), whereas the only record of this genus is a dubious fossil likely belonging to *S. rufipes* from the Early Pliocene of the Tsubusagawa Formation in Japan (Hayashi et al. 2003). The current distribution of *Sternolophus* in the Old World, i.e. without protruding into northern Asia, Europe, Tasmania and New Zealand (Nasserzadeh and Komarek 2017), which were largely covered by ice, and its absence in the fossil records from Europe and America, suggest a sensitivity of this group to climate change and glacial periods as inhibitor factors for its distribution, and also highlight the effect of eustatic changes in accelerating its dispersal in the Old World towards Australia.

## Appendix 1

**Table:** List of the specimens examined.

Species	Number of examined specimens	Collections	Total number of studied specimens	Geographical diversity of the examined specimens	Distribution of the species
*S. acutipenis*	10 (5 ♂♂, 5 ♀♀)	NMW	124	India, Thailand, Vietnam	Oriental Region
*S. angolensis*	20 (9 ♂♂, 11 ♀♀)	MNHUB, NMW, ZMUC	270	Burkina Faso, Comoros, Egypt, Guinea, Namibia, Tanzania, Togo, Zimbabwe	Afrotropical Region
*S. angustatus*	7 (5 ♂♂, 2 ♀♀)	NMW, NRM, ZMUC	50	Botswana, Namibia, South Africa, Tanzania, Zimbabwe	Eastern Afrotropical Region
*S. australis*	6 (4 ♂♂, 2 ♀♀)	FMNH, SAMA	37	Australia	Australian Region (only Australia)
*S. decens*	17 (7 ♂♂, 8 ♀♀)	NMB, NMW, HMIM	202	Iran, Oman, Pakistan, Saudi Arabia	Palaearctic and Oriental Regions (from East Africa to India)
*S. elongatus*	28 (19 ♂♂, 9 ♀♀)	ISNB, NMB, NMW, SMTD	301	Angola, Cameroon, Congo, Egypt, Eritrea, Ethiopia, Guinea, Madagascar, Saudi Arabia, Socotra Island (Yemen),	Afrotropical and Palaearctic Regions (Africa and Arabian Peninsula)
*S. immarginatus*	5 (3 ♂♂, 2 ♀♀)	SAMA, SMTD	30	Australia	Australian (only Australia)
*S. inconspicuus*	18 (11 ♂♂, 7 ♀♀)	MNHN, NNW, SMTD	234	China, India, Indonesia, Japan, Nepal, Sri Lanka, Thailand, Vietnam	Oriental Region, including southern China and Japan
*S. insulanus*	9 (6 ♂♂, 3 ♀♀)	NMW, ZMUC	37	Indonesia (Sulawesi & Papua)	Sulawesi to New Guinea
*S. jaechi*	6 (4 ♂♂, 2 ♀♀)	FMNH, NMW	13	Indonesia & Malaysia (Borneo Island)	Malay Peninsula, Borneo
*S. mandelai*	14 (7 ♂♂, 7 ♀♀)	NMW, SMTD	130	Gabon , Guinea, Namibia	Afrotropical Region
*S. marginicollis*	22 (13 ♂♂, 9 ♀♀)	NMW, SAMA, ZMUC	330	Australia, Indonesia, New Caledonia, Papua New Guinea, Philippines	Philippines and Sulawesi to New Guinea, Australia, New Caledonia and Fiji
*S. mundus*	20 (11 ♂♂, 9 ♀♀)	ISNB, MNHN, NNW, SMTD, ZMUC	416	Gabon, Kenya, Sudan, Tanzania, Uganda	Afrotropical Region
*S. prominolobus*	10 (4 ♂♂, 6 ♀♀)	HMIM, NMW, SAMA	10	Australia	Eastern Australia
*S. rufipes*	30 (17 ♂♂, 13 ♀♀)	ISNB, NNW, SMTD, AEZS	1302	China, India, Indonesia, Japan, Nepal, Philippines, Thailand, Singapore, Vietnam	Eastern Palaearctic Region, Oriental Region
*S. solieri*	33 (17 ♂♂, 13 ♀♀)	HMIM, ISNB, NMW, SMTD	635	Afghanistan, Burkina Faso, Cape Verde, Egypt, Guinea, Iran, Mali, Pakistan, Saudi Arabia, Senegal, South Sudan	Northern half of Afrotropical Region to northwestern India
*S. solitarius*	7(6 ♂♂, 1 ♀)	NMW, HMIM	12	Mauritius (Rodrigues Island), Madagascar	Madagascar, Mascarene Islands
*Hydrobius fuscicps*	4 (2 ♂♂, 2 ♀♀)	HMIM, CBSU		Iran	Holarctic
*Hydrochara flavipes*	5 (3 ♂♂, 2 ♀♀)	HMIM, CBSU		Iran	Western Palaearctic Region

## Appendix 2

**Table:** Data matrix for cladistic analysis of *Sternolophus* species based on adult morphological characters. Inapplicable data are represented by ‘?’.

	**0 0**	**0 1**	**0 2**	**0 3**	**0 4**	**0 5**	**0 6**	**0 7**	**0 0 1 8 9 0**	**1 1 1 1 1 1 2 3 4 5**	**1 1 1 1 6 7 8 9**	**2 2 2 2 2 2 0 1 2 3 4 5**	**2 2 2 2 6 7 8 9**	**3 3 3 3 3 3 0 1 2 3 4 5**	**3 3 3 3 6 7 8 9**	**4 4 4 4 4 4 0 1 2 3 4 5**	**4 4 4 4 6 7 8 9**	**5 5 5 5 5 5 0 1 2 3 4 5**	**5 5 5 5 6 7 8 9**
***Sternolophus acutipenis***	12.30	2.00	2.00	2.54	6.15	3.00	0.19	2.85	1 1 1	1 0 0 1 0	0 0 0 1	3 0 2 0 2 1	1 1 0 0	0 0 ? 0 12	? 0 ? 1	0 ? 0 0 1 0	1 1 1 1	1 1 0 ? ? ?	0 0 1 1
***S. angolensis***	11.20	1.75	1.72	2.50	6.40	2.90	0.28	3.00	1 0 1	1 0 0 1 0	1 0 0 0	? 0 3 0 2 1	1 1 1 0	0 0 ? 0 1 0	? 0 ? [01]	0 ? 0 0 1 0	0 ? ? ?	0 0 1 1 1 0	0 0 1 0
***S. angustatus***	10.70	1.75	2.20	2.30	6.10	3.00	0.30	2.80	1 1 1	0 0 0 1 1	0 0 1 0	? 1 1 0 0 1	1 0 0 0	1 ? 0 0 1 1	0 1 1 ?	? 3 1 0 0 1	1 1 1 0	0 0 1 0 0 0	1 0 0 0
***S. australis***	12.80	1.90	2.20	2.30	6.76	2.80	0.27	3.60	1 1 1	1 0 0 1 0	1 0 1 1	0 1 0 0 1 1	0 ? 0 0	0 0 ? 0 0 ?	? 0 ? 0	0 ? 0 0 01	1 0 ? ?	0 0 0 ? ? ?	0 0 1 0
***S. decens***	10.65	1.55	2.00	2.36	6.90	2.60	0.20	2.90	0 1 1	0 0 01 [01]	0 1 1 0	? 1 0 0 1 1	1 0 0 1	1 ? 1 0 1 1	0 1 0 ?	? 0 1 0 0 1	1 1 1 1	0 0 1 0 0 0	0 0 1 0
***S. elongatus***	11.60	1.80	2.30	2.30	6.40	2.70	0.20	3.00	1 0 1	0 1 0 1 1	0 0 1 0	? 1 1 0 0 1	1 0 0 0	1? 0 0 1 1	1 1 1 ?	? 2 1 0 0 1	1 1 1 0	0 0 1 0 0 0	1 0 0 0
***S. immarginatus***	12.05	1.80	2.50	2.50	6.70	3.20	0.19	2.45	1 0 1	0 0 0 1 1	1 0 1 1	1 1 2 0 1 1	1 1 1 0	0 1 ? 0 1 2	? 0 ? 0	0 ? 0 0 0 1	1 1 0 ?	0 1 1 2 1 1	0 0 1 0
***S. inconspicuus***	9.60	1.45	2.10	2.50	6.60	3.00	0.20	3.10	1 1 1	1 0 0 1 1	1 0 0 0	? 0 3 0 2 1	1 1 1 0	0 0 ? 0 1 0	? 0 ? [01]	0 ? 0 0 1 0	1 0 ? ?	0 0 1 1 0 1	0 0 1 0
***S. insulanus***	10.05	1.45	2.20	2.45	6.90	3.10	0.15	3.20	1 0 1	1 0 0 1 0	1 0 0 1	1 0 2 0 2 1	1 1 0 0	1 ? 1 0 1 0	? 0 ? 0	1 ? 0 0 1 0	1 0 ? ?	0 1 1 1 0 0	1 0 0 0
***S. jaechi***	12.40	1.80	2.40	2.60	6.90	2.90	0.22	2.80	0 1 1	1 0 0 1 0	1 0 0 1	[01] 0 3 0 2 1	1 1 0 0	0 1 ? 0 12	? 0 ? 1	1 ? 0 0 1 0	1 1 1 1	0 1 1 1 0 0	1 0 0 0
***S. mandelai***	13.05	1.95	2.05	2.30	6.70	3.10	0.32	2.70	1 0 1	0 0 0 1 1	0 1 1 0	? 1 1 0 0 1	1 0 0 0	1 ? 1 0 1 1	0 1 1 ?	? 2 1 0 0 1	1 1 1 0	0 0 1 0 0 0	1 0 0 0
***S. marginicollis***	11.40	1.80	2.75	2.60	6.30	3.10	0.19	3.00	1 1 1	1 0 0 1 0	1 0 0 1	[23] 0 2 0 2 1	1 1 0 0	0 0 ? 0 1 1	0 0 ? [01]	1 ? 0 0 1 0	1 0 ? ?	0 1 1 2 0 0	0 0 1 0
***S. mundus***	15.30	2.25	1.90	2.50	6.80	2.80	0.30	2.90	1 0 1	1 0 0 1 0	1 0 0 0	? 0 3 0 2 1	1 1 1 0	0 1 ? 0 1 0	? 0 ? 1	1 ? 0 0 1 0	1 1 1 0	0 0 1 2 1 0	0 0 1 0
***S. prominolobus***	12.45	2.00	2.05	2.25	6.20	2.90	0.15	2.90	0 1 1	1 0 01 0	1 0 0 1	2 0 2 0 21	1 1 0 0	0 1 ? 0 1 1	1 0 ? 0	0 ? 0 0 1 0	1 0 ? ?	0 0 1 1 0 1	1 0 0 0
***S. rufipes***	10.40	1.70	2.10	2.43	6.12	3.05	0.25	3.20	1 1 1	0 1 1 1 1	0 1 1 0	? 1 1 0 0 1	1 0 0 0	1 ? 1 0 1 1	0 1 1 ?	? 3 1 0 0 1	1 1 1 0	0 0 1 0 0 0	1 0 0 0
***S. solieri***	10.30	1.55	2.17	2.30	6.64	2.90	0.29	3.10	1 0 1	0 1 1 1 1	0 1 1 0	? 1 1 0 0 1	1 0 0 0	1 ? 1 0 1 1	0 1 0 ?	? 1 1 0 0 1	1 1 1 0	0 0 1 0 0 0	1 0 0 0
***S. solitarius***	12.45	2.00	2.00	2.50	6.22	3.20	0.20	2.85	1 1 1	1 0 0 1 0	1 0 0 0	? 0 2 0 2 1	1 1 1 0	0 1 ? 0 1 0	? 0 ? 0	0 ? 0 0 1 1	1 1 1 0	0 0 1 1 1 0	0 0 1 0
***Hydrochara flavipes***	15.50	2.70	2.20	2.30	5.70	2.90	0.19	3.40	1 1 0	? 1 1 0 1	0 1 1 0	? 1 1 1 ? 0	? ? 0 0	0 0 ? 1 ? ?	? 0 ? 0	0 ? 1 1 0 1	1 1 1 1	0 0 0 ? ? ?	1 1 2 0
